# Shared and Unique Signals of High-Altitude Adaptation in Geographically Distinct Tibetan Populations

**DOI:** 10.1371/journal.pone.0088252

**Published:** 2014-03-18

**Authors:** Tana Wuren, Tatum S. Simonson, Ga Qin, Jinchuan Xing, Chad D. Huff, David J. Witherspoon, Lynn B. Jorde, Ri-Li Ge

**Affiliations:** 1 Research Center for High-Altitude Medicine, Qinghai University Medical School, Xining, Qinghai, People's Republic of China; 2 Division of Physiology, Department of Medicine, University of California San Diego, La Jolla, California, United States of America; 3 Department of Human Genetics, University of Utah School of Medicine, Salt Lake City, Utah, United States of America; University of Utah, United States of America

## Abstract

Recent studies have used a variety of analytical methods to identify genes targeted by selection in high-altitude populations located throughout the Tibetan Plateau. Despite differences in analytic strategies and sample location, hypoxia-related genes, including *EPAS1* and *EGLN1*, were identified in multiple studies. By applying the same analytic methods to genome-wide SNP information used in our previous study of a Tibetan population (n = 31) from the township of Maduo, located in the northeastern corner of the Qinghai-Tibetan Plateau (4200 m), we have identified common targets of natural selection in a second geographically and linguistically distinct Tibetan population (n = 46) in the Tuo Tuo River township (4500 m). Our analyses provide evidence for natural selection based on iHS and XP-EHH signals in both populations at the p<0.02 significance level for *EPAS1*, *EGLN1*, *HMOX2*, and *CYP17A1* and for *PKLR*, *HFE*, and *HBB* and *HBG2*, which have also been reported in other studies. We highlight differences (i.e., stratification and admixture) in the two distinct Tibetan groups examined here and report selection candidate genes common to both groups. These findings should be considered in the prioritization of selection candidate genes in future genetic studies in Tibet.

## Introduction

Native highlanders have specific traits that enable their survival despite the stress imposed by decreased oxygen availability at altitude. Several regions of the genome, including those that harbor hypoxia-sensing and -regulated genes, were recently identified as putative targets for high-altitude adaptation in Tibetans [1]–[11]. Two of these regions contain genes involved in hypoxia sensing and response: the *EPAS1* gene, which encodes the hypoxia inducible factor (HIF)-2α subunit, and the *EGLN1*gene, which encodes a proline hydroxylase, PHD2, that regulates hypoxia-induced factors in an oxygen-dependent manner. Variation in the *EPAS1* region is associated with hemoglobin concentration ([Hb]) in Tibetan populations examined in two separate studies [2], [10], and haplotypes at loci containing *EGLN1* and *PPARA* (peroxisome proliferator activated receptor alpha) are associated with [Hb] in Tibetans from the Maduo township in the northeast section of the Qinghai-Tibetan Plateau [7] and in a sample of Tibetans located throughout the Plateau [12]. *EPAS1* and *PPARA* haplotypes in Tibetans from the Tuo Tuo River examined here are associated with elevated serum lactate and free fatty acid concentrations, respectively, providing further support for important biological roles [13]. In addition to these phenotype-associated selection targets, many other genes have been reported as strong targets of selection in other studies and are likely associated with additional adaptive traits in Tibetan populations [6]. Here we highlight selection signals identified at the p<0.02 empirical significance level in previously examined Maduo [7] and Tuo Tuo River [13] Tibetan populations using the same analytical strategies.

Tibetans inhabit a vast area of the Qinghai-Tibetan Plateau, which spans approximately 1.5 million square km (0.96 million square miles). At least three major Tibetan dialects are spoken among these geographically distinct groups (Amdo, U-Tsang, and Kham in the northeastern, southwestern, and southeastern regions of the Plateau, respectively [14]), suggesting potential genetic isolation among different Tibetan populations. The demographic history of these populations is, however, highly debated. Archaeological evidence suggests that the ancestors of present-day Tibetan groups migrated to the Qinghai-Tibetan Plateau at various times, ranging from 25,000 to 5,000 years ago [15]. Patterns of genome-wide SNPs support a single-route migration into this region [8], although analyses of mitochondrial DNA variation suggest that different migrations, dating to pre- and post- Last Glacial Maximum, contributed to genetic variation observed among present-day inhabitants [16], [17]. Neighboring populations have also mixed with various Tibetan groups located on the periphery of the plateau, contributing to present-day levels of variation in these regions [8].

While various studies have focused on a few select candidate gene regions [1], [4], [5], many genetic loci have probably been targeted by selection in high-altitude populations (see review [6]). In order to evaluate whether our previously reported selection targets [7] are significant in a different Tibetan group [13], we carried out genome-wide SNP-based selection scans in a linguistically distinct population from a different region of the Qinghai-Tibetan Plateau. Several of the same haplotypes exhibit extreme signals of selection in this second population, highlighting genomic regions that warrant further investigation in genetic studies of high-altitude adaptation in Tibet. We also determined which regions with extreme selection signals contain hypoxia-associated microRNAs. We report differences in the prevalence of admixture and prevalence of mitochondrial haplogroups in two distinct Tibetan groups, highlighting the value of studying different Tibetan groups and accounting for varied genetic backgrounds.

## Results

### Stratification of Tibetan populations

We used pair-wise allele sharing distances to calculate F_ST_ [18] among Tibetan populations and neighboring Asian groups. The two Tibetan populations exhibit the least amount of genetic differentiation from each other (F_ST_ = 0.004) compared to other Asian populations (HapMap Chinese [CHB] and Japanese [JPT] [19], and Buryat and Deedu Mongolians [20], [21]. Tuo Tuo River and Maduo Tibetans are similarly differentiated from the Hap Map CHB (F_ST_ = 0.014 and 0.012, respectively), which are commonly used for genetic comparisons (Table S1).

**Figure 1:**
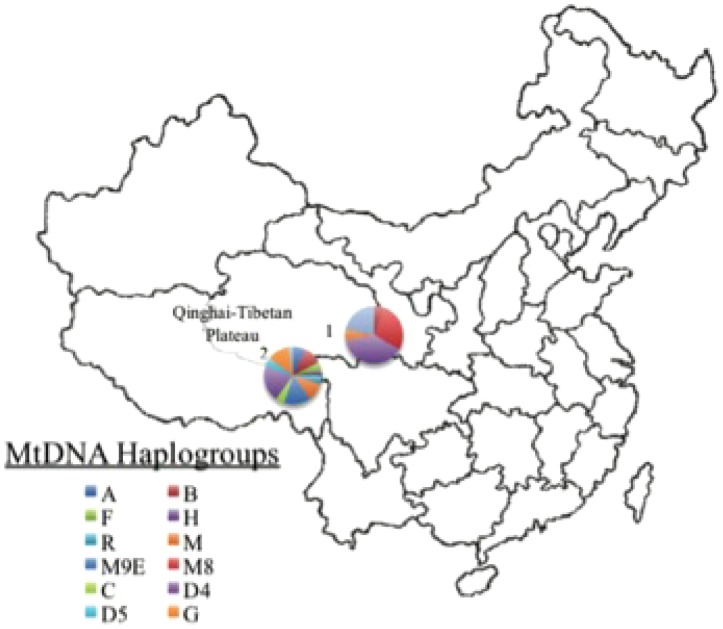
Map of two sample locations and mtDNA haplogroup frequencies from Maduo (represented by the furthest northeast) and Tuo Tuo River regions. Numbers 1 and 2 represent sample locations for Maduo [7] and Tuo Tuo River sample locations, respectively.

To determine the extent of population structure among Tibetans and neighboring populations, we used the program Admixture [22] to examine the group-specific proportion of each individual's genome (Figure 1). Each of the Tibetan populations, as well as Buryat Mongolians, Deedu Mongolians, HapMap CHB, and HapMap JPT populations, form a distinct group. As previously reported, Maduo Tibetans exhibit components associated with Han Chinese (CHB), suggesting either recent admixture or may reflect different founder populations in this group [7]. Individuals from the Tuo Tuo River population do not, however, exhibit this signal, likely reflecting the more insulated geographic location of this group (Figure 1).

### High-altitude selection candidate genes identified in two distinct Tibetan populations

We employed the same analytic strategies (iHS [23] and XP-EHH [24] statistics) used in our previous study of a Tibetan high-altitude adaptation to identify mutual targets of selection in a second linguistically distinct Tibetan population. By applying the same analytical methods in both populations, we provide further evidence for positive selection in each of two distinct Tibetan populations at the p<0.02 significance level for *EPAS1*, *EGLN1*, *HMOX2*, and *CYP17A1* gene regions (Table 1) and further support for adaptation involving *PKLR*, *HFE*, and *HBB* and *HBG2* genes identified in other recent studies. The first population described [7] is a group of Qinghai-Tibetans from Maduo who speak the Amdo Tibetan dialect (one of the three major Tibetan dialects spoken on the Qinghai-Tibetan Plateau); the second population examined, who speak the Kham dialect, are from the Tuo Tuo River area [13] (Figure 1). In order to identify high-altitude specific selection candidates, genomic regions with iHS significance levels at p<0.02 identified in our other Asian populations were excluded from analysis as described previously [7]. Of the selection candidate genes reported in our previous study of Maduo Tibetans [7], seven were identified at the p<0.02 significance level in Tuo Tuo River Tibetans and/or have been reported as strong selection candidates in other studies of Tibetan adaptation to altitude (Table 1).

**Table 1:** Selection signals identified at the empirical top 2% significance level in Tibetans from Maduo (as reported in Simonson et al. 2010) and Tuo Tuo River Tibetans and/or reported in other studies of human adaptation to high altitude as referenced.

Gene	Chromosome	200 kb Bin	P value	Selection scan	References for candidate genes
*EGLN1*	Chr1	1147	1.23E-03	XP-EHH Maduo	[3], [7], [8], [9], [10], [11], [12]
		1148	1.54E-04	XP-EHH Maduo	
			9.83E-03	iHS Maduo	
			9.45E-03	iHS Tuo Tuo River	
*EPAS1*	Chr2	231	1.54E-03	XP-EHH Maduo	[1], [3], [7], [8], [9], [10]
		232	1.03E-02	XP-EHH Maduo	
			4.09E-03	iHS Tuo Tuo River	
*PPARA*	Chr22	224	9.00E-03	iHS Maduo	[7], [25]
*CYP17A1*	Chr10	522	7.09E-03	iHS Maduo	[7]
			1.40E-02	iHS Tuo Tuo River	
*HMOX2*	Chr16	22	1.29E-03	iHS Maduo	[7], [11]
			7.08E-04	iHS Tuo Tuo River	
			1.24E-03	XP-EHH Tuo Tuo River	
*PKLR*	Chr1	767	4.33E-03	iHS Tuo Tuo River	[10]
			2.00E-02	iHS Maduo	[7]
*HBB* and *HBG2*	Chr11	26	0.0158		[7], [10]
*HFE*	Chr6	131	1.37E-02	iHS Tuo Tuo River	[10]

The 200 kb bin refers to the genomic position on the chromosome listed in the second column (positions based on Hg18) from which the selection signal emanates. The empirical p value of this region, which contains the selection candidate gene, is based on the selection analysis performed in the population specified.

### Genotype-phenotype analysis of hemoglobin concentration

The putatively adaptive *EPAS1* genomic region is associated with [Hb] in two independent studies of high-altitude adaptation in Tibetans [2], [10]. While an extreme signal of selection near the *EPAS1* gene was detected in both the Maduo and Tuo Tuo River Tibetan populations, the *EPAS1* haplotype did not exhibit an association with [Hb] in Maduo Tibetans as previously reported [7], nor was there a significant association in Tuo Tuo River Tibetans (n = 36) examined here (Table S3; Figure S1). It is likely that our modest sample size yields reduced power to detect phenotype-genotype associations (Figure S2).

**Figure 2:**
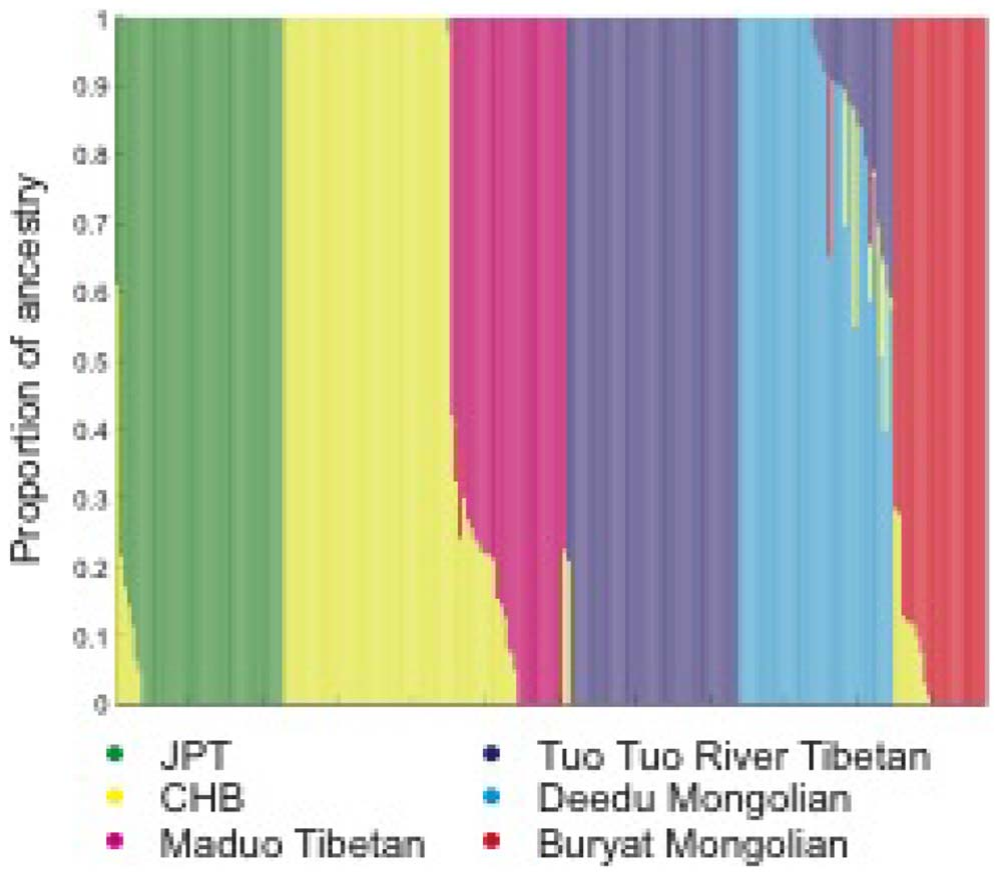
Population structure of Tibetans and neighboring populations. Individual grouping inferred by Admixture with k = 6, arranged by population. Each vertical bar represents an individual's genome. The colors correspond to the proportion of an individual's ancestry derived from one of the k groups.

In our previous study of Maduo Tibetans, we identified associations between *EGLN1* and *PPARA* selected regions and [Hb] [7]. There is no association between the *EGLN1* and *PPARA* haplotypes and [Hb] in the 36 Tuo Tuo River Tibetans examined here (Table S3; Figure S1). We speculate that genetic heterogeneity may influence genotype-phenotype relationships as a result of the genetic admixture that was detected in Maduo but not Tuo Tuo River Tibetans (Figure 2). It will be important to test, in a larger sample, whether admixture differences underlie reduced power to detect a signal in this modest sample (Figure S2).

### Non-protein coding regions of the genome highlighted in both Tibetan groups

In order to determine whether non-genic microRNAs not previously examined as *a priori* candidates are contained within our top selection candidates, we examined the intersection between the top two percent of iHS and XP-EHH selection candidate regions and genomic locations that contain any of 54 hypoxia-related miRNAs [25]. Interestingly, the *PPARA* selection candidate region identified in our previous study contains a hypoxia-associated miRNA (Table S2) that may be involved in high-altitude response, although this region was not identified as a top selection candidate in the Tuo Tuo River Tibetans. A recent report of adaptation in Ethiopian highlanders also reports an extreme signal of selection near *PPARA* [26], although it is unclear whether the hypoxia-related miRNA in this region is involved in adaptation of either Tibetan or Ethiopian highland groups.

### Mitochondrial DNA analyses highlight variation in Maduo and Tuo Tuo River Tibetan populations

Entire mtDNA control region sequence and genotyping of present-day inhabitants of the Tibetan Plateau indicate distinct patterns of variation among Tibetan groups [16], [27]. In order to determine whether the two Tibetan populations exhibit differences in mitochondrial DNA (mtDNA) haplogroups, we genotyped 12 haplotype-defining SNPs in18 and 40 individuals from Maduo and Tuo Tuo River (Figure 1), respectively. Consistent with previously reported frequencies for Tibeto-Burman groups, the most common major mtDNA haplogroup is M (94% and 67.5% for Maduo and TTR, respectively), although a small proportion of haplogroup N is also detected in our samples. M9, which is found at elevated frequency at high-altitude inhabitants in Tibet and India [28], is predominant in both Maduo and Tuo Tuo River populations (Table 2). The sub-M haplogroups D4, M9E, and M8 are most prevalent among Maduo Tibetans, whereas M9E and G are the most common mtDNA haplogroups within the TTR sample. Haplogroup A, which exhibits greater diversity in the southern region of the Qinghai-Tibetan Plateau [17], is detected among Tibetan inhabitants of the Tuo Tuo River area (8%) and Maduo Tibetans who reside further north (7%). Haplogroup B, a major group common among Native American populations, is also present in Tuo Tuo River Tibetans (TTR: 10%). The power to detect mtDNA variants at marginal frequencies (<10%) is limited in our modest sample of mtDNA from the Maduo population, and additional sample collection will be necessary to place these samples in the context of global mtDNA variation and determine precise genetic relationships among these populations.

**Table 2:** Mitochondrial DNA haplogroup allele frequencies in Maduo and Tuo Tuo River Tibetans.

MtDNA Haplogroup	M	M8	M9E	C	D4	D5	G	N	A	B	F	H	R
TTR Tibetan	0.10		0.15	0.05	0.20	0.05	0.13	0.03	0.08	0.10	0.05	0.03	0.05
Maduo Tibetan		0.22	0.28		0.39		0.06		0.06				

## Discussion

Recent reports of high-altitude adaptation in Tibetans are based on a range of analytical methods applied to different Tibetan populations located throughout the Qinghai-Tibetan Plateau [1], [4]–[6]. While some of the selection candidate genes reported by these studies are the same, there are also selection targets unique to each study. This could be due in part to differences in analytical methods, which range from sequence-based examination of allele-frequency differences between Tibetan and non-Tibetan groups to genome-wide analyses of extended SNP haplotype variation [1], [4]–[6].

Highly differentiated SNPs in the *EPAS1* gene region are related to relatively lower [Hb] in two independent studies of high-altitude adaptation in Tibet [2], [10], and recent sequencing efforts have identified two variants in the first exon of *EGLN1* that are highly differentiated in Tibetans [12], [29]. Another study of the *EGLN1* region shows that two polymorphisms within the first intron are found at elevated frequency (71%) in a high-altitude population from India and are associated with *EGLN1* expression and high-altitude pulmonary edema (HAPE) in this population [30]. These SNPs are in complete linkage disequilibrium (r^2^ = 1.0) with the putatively adaptive selected haplotypes identified in both of the Tibetan populations examined here, providing additional evidence for an adaptive role of *EGLN1* across the Qinghai-Tibetan Plateau. Interestingly, *EGLN1* was identified as a target of selection in Andean highlanders, although it is unclear whether the putatively adaptive variants are the same as those reported in Tibetan highlanders [3], [31]. A study of highland Daghestani populations also indicates highly differentiated intronic SNPs in a well-conserved region of *EGLN1* [32].

While *EPAS1* [2], [3], [7], [9]–[11], *EGLN1* [3], [7], [9]–[11], [31], and *PPARA* [7], [26] have been identified as selection candidates in one or more studies of altitude adaptation and/or are associated with Tibetan putatively adaptive phenotypes, several other selection candidates have also been identified in multiple studies. The *HMOX2* gene, a heme oxygenase involved in HIF-independent hypoxia sensing, was a top selection candidate in our Maduo and Tuo Tuo River populations and was also reported as a selection candidate in a pooled sample of 50 Tibetan samples collected from various regions of the Qinghai-Tibetan Plateau [11]. Furthermore, *PKLR* and *HBB/HBG2*, respectively identified in the Tuo Tuo River and Maduo Tibetan populations at the p<0.02 level, were also reported as top selection candidates in an independent examination of genetic variation in 50 Tibetan exomes [10]. Variants in the *CYP17A1* gene, contained within a top selection candidate region identified in both Maduo and Tuo Tuo River populations, are associated with hypertension in European and Asian populations [33]–[35]. While these selection candidates have not been associated with the phenotypes measured thus far, they should be considered as strong candidates for future studies of genetic and physiological adaptations.

Considering the lack of differentiation detected through analysis of the protein-coding regions of Han Chinese and Tibetan samples [10], it is also possible that many genetic targets of selection are in non-coding, regulatory regions of the genome. Our analyses suggest natural selection on a miRNA near the *PPARA* gene, highlighting regulatory variation as a potential factor underlying adaptive advantages within Tibetan genomes.

Differences among studies published to date could also result from variation in the genetic background of Tibetan groups. Such factors could influence the extent to which selection signals are captured (the early stages of a selection sweep versus those that are fixed or nearly fixed in the population) or the potential for selection events to occur in different groups. In order to better understand differences in Tibetan adaptation to altitude, it will be necessary to fully characterize population histories in various Tibetan groups, determine the precise functional variants and timing of selection events, and evaluate additional phenotypic differences related to adaptive functional variants.

### Conclusions

Selection and association signals identified in Tibetan populations may be influenced by the demographic history of these groups and by the choice of analytic methods. Despite genetic heterogeneity between Tibetan groups as shown here, several putatively adaptive genetic variants are common to Maduo and Tuo Tuo River Tibetans. Further genetic and phenotypic characterizations of physiological traits (e.g., oxygen transport, maternal/fetal responses during pregnancy) are required to determine if the remaining selection targets highlighted here are of biological significance, whether they are related to or independent of [Hb], and how combinations of such factors influence the overall physiology of native highlanders living in the Tibetan Plateau.

## Materials and Methods

### DNA sample collection

We extracted DNA from whole blood samples obtained from 85 highland natives (non-smokers, no chronic diseases) residing in the Tuo Tuo River township in Qinghai Province (∼4,500 m).

### Ethics Statement

Participants provided written agreement as indicated by a signature or mark on a sheet of paper that described the study in their native language. Consent was obtained for all participants, and this study was approved by the Institutional Review Board at the High Altitude Medical Research Institute (Xining, Qinghai, People's Republic of China).

### SNP genotyping

We employed the Affymetrix 6.0 SNP Array technology (>900,000 SNPs) to genotype 70 DNA samples at Capital Bio Corporation (Beijing, China). Default parameters for the Birdseed algorithm (version 2) were used to determine genotypes for all samples (Affymetrix, Santa Clara, CA, USA). Genotypic data were analyzed using the Affymetrix Genotyping Console 3.1 (Affymetrix) and included all autosomes but excluded the X and Y chromosomes and the mitochondrial genome.

### Estimates of relatedness

We attempted to exclude all first-degree relatives who visited the clinic from our study. We used the ERSA software package [36] to determine genetic relatedness among all individuals examined and excluded one member of any pair exhibiting relatedness closer than second cousins. Based on these criteria, a total of 46 unrelated individuals were included in the analyses. The genotype data of the 46 individuals are available at http://jorde-lab.genetics.utah.edu/ under published data.

### Principal components analysis

We performed principal components analysis on genetic distances as previously reported [21]. This analysis included two Tibetan populations from distinct regions and other Asian groups such as the HapMap CHB and JPT populations (CHB = Chinese in Beijing, China; JPT = Japanese in Tokyo, Japan). The CEU (U.S. Utah residents with ancestry from northern and western Europe) and YRI (YRI = Yoruba in Ibadan, Nigeria) HapMap populations provide context for the patterns of variation observed among these populations [19].

### Functional candidate gene list

We had previously generated a list of genes likely related to high-altitude adaptation based on Gene Ontology and Panther Pathway categories as described in [7] and examined the intersection between these candidates and selection candidate genes identified at the p<0.02 level. Potential candidate genes identified in the mitochondrial genome and on the X chromosome were not considered for this study.

### Admixture analysis

A model-based algorithm implemented in *ADMIXTURE* [22] was used to determine the genetic ancestries of each individual in a given number of populations without using information about population designation. To eliminate the effects of SNPs that are in linkage disequilibrium (r^2^ = 1.0), we first filtered out SNPs that had r^2^>0.2 within 100 kb using PLINK [37], as recommended by the authors of *ADMIXTURE*.

### Selection analyses

We performed the iHS [23] and XP-EHH [24] tests of selection on phased data estimated by the Beagle software package as previously described [7]. These tests are based on extended haplotype homozygosity and measure the reduction in haplotype diversity based on the probability that, as distance from a focal SNP increases, two extended haplotypes are the same. XP-EHH compares across populations (in this case, Tuo Tuo River Tibetans and HapMap Han Chinese and Japanese); iHS compares within-population profiles based on the focal SNP's ancestral state (derived or ancestral).

We focused on the selection candidate genes contained within 200 kb regions significant at the p<0.02 level in either test. We further excluded regions where the iHS test was significant at this level in neighboring populations as previously described [7].

### Phenotype collection

Hemoglobin concentration, hematocrit, and percent oxygen saturation were determined from venous blood samples using the Mindray Hematology Analyzer (BC-2300, Shenzhen, People's Republic of China) and the Pulse Oximeter (Ohmeda 3700 Pulse Oximeter, Datex-Ohmeda, Boulder, Colorado, USA), respectively.

### Genotype-phenotype association

We identified the putatively advantageous haplotypes as previously described [7] and tested whether the three-SNP allele haplotype exhibiting the most extreme iHS scores within each 200 kb genomic region were associated with [Hb] in each population and both populations combined. Stepwise linear regression (MATLAB R2010a) was used to detect significant relationships between these genotypes and [Hb].

## Supporting Information

Figure S1 **The relationships between** ***EPAS1*** **,** ***EGLN1*** **, and** ***PPARA*** **haplotypes and [Hb] for Tuo Tuo River Tibetans (shown in open circles) and Maduo Tibetans (closed circles).** The number of *PPARA* haplotype copies, previously associated with [Hb] in Maduo Tibetans (p<0.0005; Simonson et al. 2010), is associated with [Hb] when data from both populations are combined (p<0.02).
(TIF)

Figure S2 **Statistical power to detect an association of [Hb] with a haplotype.** Simulated data sets were constructed with varying sample size (*n* = 30–500), assuming that the putatively selected haplotype at one locus decreases [Hb] by *e* g/dl when present in two copies and *e*/2 if present in one copy (additive model, *e* = 0.5, 1.0, 2.0). [Hb] was simulated as a normally-distributed variable with mean 19.6 and standard deviation of 1.6 g/dl, as observed in the Tuo Tuo River sample, and the effect of adaptive haplotype copies was added to that variate for each individual. The frequency *f* of the adaptive haplotype was set at 0.65, 0.75 or 0.85, per the legend. To mirror the actual tests performed, haplotypes with no effect on [Hb] were simulated for two additional loci (haplotype frequency of 0.65 for both). Genotypes were assigned in Hardy-Weinberg equilibrium. Ages were assigned from a normal distribution, mean 37 years and standard deviation 11.5, then truncated to the range of 18–68, mirroring the observed distribution. Sex was assigned randomly with a 50/50 ratio. Multiple stepwise linear regression was performed using the five simulated predictors: age, sex and haplotype copies at three loci (as used in Table S3). Power to detect a significant association of [Hb] with the simulated adaptive haplotype was estimated as the fraction of 1000 iterations for each parameter set that yielded a significant result at the alpha = 0.5 level. Effect size *e* has the largest impact on statistical power. Haplotype frequency has a modest influence (Tuo Tuo River *EGLN1*, *EPAS1*, and *PPARA* frequencies = 0.68, 0.81, and 0.77, respectively). Considering our modest sample size, it will be necessary to collect more data from the Tuo Tuo River population to achieve greater power to detect genotype-phenotype associations.
(TIF)

Table S1 **F_ST_ for Tibetans and neighboring Asian populations examined.**
(DOCX)

Table S2 **Intersection of selection candidate regions and hypoxia-related miRNAs.**
(DOCX)

Table S3 **Multiple stepwise linear regression including age, sex, and three haplotypes previously identified as selection candidates in Tibetans (** ***EGLN1*** **,** ***PPARA*** **, and** ***EPAS1*** **) in Tuo Tuo River Tibetans.** Gender was the only significant (p<0.01) predictor in this analysis (F = 14.82, P<0.0005).
(DOCX)

## References

[1] Beall CM (2011) Genetic Changes in Tibet. High Altitude Medicine & Biology 12: 101–102. 21718152 10.1089/ham.2011.1007

[2] Beall CM, Cavalleri GL, Deng L, Elston RC, Gao Y, et al (2010) Natural selection on EPAS1 (HIF2a) associated with low hemoglobin concentration in Tibetan highlanders. Proceedings of the National Academy of Sciences 107: 11459–11464. 10.1073/pnas.1002443107 PMC2895075 20534544

[3] Bigham A, Bauchet M, Pinto D, Mao X, Akey JM, et al (2010) Identifying Signatures of Natural Selection in Tibetan and Andean Populations Using Dense Genome Scan Data. PLoS Genet 6: e1001116. 20838600 10.1371/journal.pgen.1001116 PMC2936536

[4] MacInnis MJ, Rupert JL (2011) 'ome on the Range: Altitude Adaptation, Positive Selection, and Himalayan Genomics. High Altitude Medicine & Biology 12: 133–139. 21718161 10.1089/ham.2010.1090

[5] Scheinfeldt L, Tishkoff S (2010) Living the high life: high-altitude adaptation. Genome Biology 11: 133. 20979669 10.1186/gb-2010-11-9-133 PMC2965377

[6] Simonson TS, McClain DA, Jorde LB, Prchal JT (2012) Genetic determinants of Tibetan high-altitude adaptation. Hum Genet 131: 527–533. 22068265 10.1007/s00439-011-1109-3

[7] Simonson TS, Yang Y, Huff CD, Yun H, Qin G, et al (2010) Genetic Evidence for High-Altitude Adaptation in Tibet. Science 329: 72–75. 20466884 10.1126/science.1189406

[8] Wang B, Zhang Y-B, Zhang F, Lin H, Wang X, et al (2011) On the Origin of Tibetans and Their Genetic Basis in Adapting High-Altitude Environments. PLoS One 6: e17002. 21386899 10.1371/journal.pone.0017002 PMC3046130

[9] Xu S, Li S, Yang Y, Tan J, Lou H, et al (2011) A Genome-Wide Search for Signals of High Altitude Adaptation in Tibetans. Molecular Biology and Evolution 28 (2) 1003–1011. 20961960 10.1093/molbev/msq277

[10] Yi X, Liang Y, Huerta-Sanchez E, Jin X, Cuo ZXP, et al (2010) Sequencing of 50 Human Exomes Reveals Adaptation to High Altitude. Science 329: 75–78. 20595611 10.1126/science.1190371 PMC3711608

[11] Peng Y, Yang Z, Zhang H, Cui C, Qi X, et al (2011) Genetic Variations in Tibetan Populations and High-Altitude Adaptation at the Himalayas. Molecular Biology and Evolution 28: 1075–1081. 21030426 10.1093/molbev/msq290

[12] Xiang K, Ouzhuluobu, Peng Y, Yang Z, Zhang X, et al (2013) Identification of a Tibetan-specific mutation in the hypoxic gene EGLN1 and its contribution to high-altitude adaptation. Mol Biol Evol 30: 1889–1898. 23666208 10.1093/molbev/mst090

[13] Ge RL, Simonson TS, Cooksey RC, Tanna U, Qin G, et al (2012) Metabolic insight into mechanisms of high-altitude adaptation in Tibetans. Mol Genet Metab 106: 244–247. 22503288 10.1016/j.ymgme.2012.03.003 PMC3437309

[14] Qingying C (1990) Tibetan Tribe In China. Qinghai Academy Of Social Sciences Tibetan Culture Research Institute ISBN7-80057-043-6.

[15] Aldenderfer M (2011) Peopling the Tibetan Plateau: Insights from Archaeology. High Altitude Medicine & Biology 12: 141–147. 21718162 10.1089/ham.2010.1094

[16] Qi X, Cui C, Peng Y, Zhang X, Yang Z, et al (2013) Genetic evidence of paleolithic colonization and neolithic expansion of modern humans on the tibetan plateau. Mol Biol Evol 30: 1761–1778. 23682168 10.1093/molbev/mst093

[17] Qin Z, Yang Y, Kang L, Yan S, Cho K, et al (2010) A mitochondrial revelation of early human migrations to the Tibetan Plateau before and after the last glacial maximum. American Journal of Physical Anthropology 143: 555–569. 20623602 10.1002/ajpa.21350

[18] Cockerham CC, Weir BS (1984) Covariances of relatives stemming from a population undergoing mixed self and random mating. Biometrics 40: 157–164. 6733226

[19] HapMap Consortium (2007) A second generation human haplotype map of over 3.1 million SNPs. Nature 449: 851–861. 17943122 10.1038/nature06258 PMC2689609

[20] Xing J, Watkins WS, Witherspoon DJ, Zhang Y, Guthery SL, et al (2009) Fine-scaled human genetic structure revealed by SNP microarrays. Genome Research 19: 815–825. 19411602 10.1101/gr.085589.108 PMC2675970

[21] Xing J, Wuren T, Simonson TS, Watkins WS, Witherspoon DJ, et al (2013) Genomic analysis of natural selection and phenotypic variation in high-altitude mongolians. PLoS Genet 9: e1003634. 23874230 10.1371/journal.pgen.1003634 PMC3715426

[22] Alexander DH, Novembre J, Lange K (2009) Fast model-based estimation of ancestry in unrelated individuals. Genome Research 19: 1655–1664. 19648217 10.1101/gr.094052.109 PMC2752134

[23] Voight BF, Kudaravalli S, Wen X, Pritchard JK (2006) A Map of Recent Positive Selection in the Human Genome. PLoS Biology 4: e72. 16494531 10.1371/journal.pbio.0040072 PMC1382018

[24] Sabeti PC, Varilly P, Fry B, Lohmueller J, Hostetter E, et al (2007) Genome-wide detection and characterization of positive selection in human populations. Nature 449: 913–918. 17943131 10.1038/nature06250 PMC2687721

[25] Kulshreshtha R, Davuluri RV, Calin GA, Ivan M (2008) A microRNA component of the hypoxic response. Cell Death Differ 15: 667–671. 18219318 10.1038/sj.cdd.4402310

[26] Scheinfeldt LB, Soi S, Thompson S, Ranciaro A, Woldemeskel D, et al (2012) Genetic adaptation to high altitude in the Ethiopian highlands. Genome Biol 13: R1. 22264333 10.1186/gb-2012-13-1-r1 PMC3334582

[27] Zhao M, Kong Q-P, Wang H-W, Peng M-S, Xie X-D, et al (2009) Mitochondrial genome evidence reveals successful Late Paleolithic settlement on the Tibetan Plateau. Proceedings of the National Academy of Sciences 106: 21230–21235. 10.1073/pnas.0907844106 PMC2795552 19955425

[28] Ji F, Sharpley MS, Derbeneva O, Alves LS, Qian P, et al (2012) Mitochondrial DNA variant associated with Leber hereditary optic neuropathy and high-altitude Tibetans. Proc Natl Acad Sci U S A 109: 7391–7396. 22517755 10.1073/pnas.1202484109 PMC3358837

[29] Lorenzo F, Swierczek S, Huff C, Prchal JT (2011) The Tibetan PHD2 polymorphism Asp4Glu is associated with Hypersensitivity of Erythroid Progenitors to EPO and Upregulation of HIF-1 Regulated Genes hexokinase (*HK1*) and glucose transporter 1 (*SLC2A*/*GLUT1*). Abstract.

[30] Aggarwal S, Negi S, Jha P, Singh PK, Stobdan T, et al (2010) EGLN1 involvement in high-altitude adaptation revealed through genetic analysis of extreme constitution types defined in Ayurveda. Proceedings of the National Academy of Sciences 107: 18961–18966. 10.1073/pnas.1006108107 PMC2973881 20956315

[31] Bigham AW, Mao X, Brutsaert T, Wilson MJ, Julian CG, et al (2009) Identifying positive selection candidate loci for high-altitude adaptation in Andean populations. Human Genomics 4: 79–90. 20038496 10.1186/1479-7364-4-2-79 PMC2857381

[32] Pagani L, Ayub Q, MacArthur DG, Xue Y, Baillie JK, et al (2012) High altitude adaptation in Daghestani populations from the Caucasus. Hum Genet 131: 423–433. 21904933 10.1007/s00439-011-1084-8 PMC3312735

[33] Hong KW, Jin HS, Lim JE, Kim S, Go MJ, et al (2010) Recapitulation of two genomewide association studies on blood pressure and essential hypertension in the Korean population. J Hum Genet 55: 336–341. 20414254 10.1038/jhg.2010.31

[34] Levy D, Ehret GB, Rice K, Verwoert GC, Launer LJ, et al (2009) Genome-wide association study of blood pressure and hypertension. Nat Genet 41: 677–687. 19430479 10.1038/ng.384 PMC2998712

[35] Takeuchi F, Isono M, Katsuya T, Yamamoto K, Yokota M, et al (2010) Blood pressure and hypertension are associated with 7 loci in the Japanese population. Circulation 121: 2302–2309. 20479155 10.1161/CIRCULATIONAHA.109.904664

[36] Huff CD, Witherspoon DJ, Simonson TS, Xing J, Watkins WS, et al (2011) Maximum-likelihood estimation of recent shared ancestry (ERSA). Genome Res 21: 768–774. 21324875 10.1101/gr.115972.110 PMC3083094

[37] Purcell S, Neale B, Todd-Brown K, Thomas L, Ferreira MAR, et al (2007) PLINK: A Tool Set for Whole-Genome Association and Population-Based Linkage Analyses. The American Journal of Human Genetics 81: 559–575. 17701901 10.1086/519795 PMC1950838

