# Supplementary figures and images for: Shared and Unique Signals of High-Altitude Adaptation in Geographically Distinct Tibetan Populations
Source: PLoS One. 2014 Mar 18;9(3):e88252. doi: 10.1371/journal.pone.0088252 (PMC3958363; doi:10.1371/journal.pone.0088252)

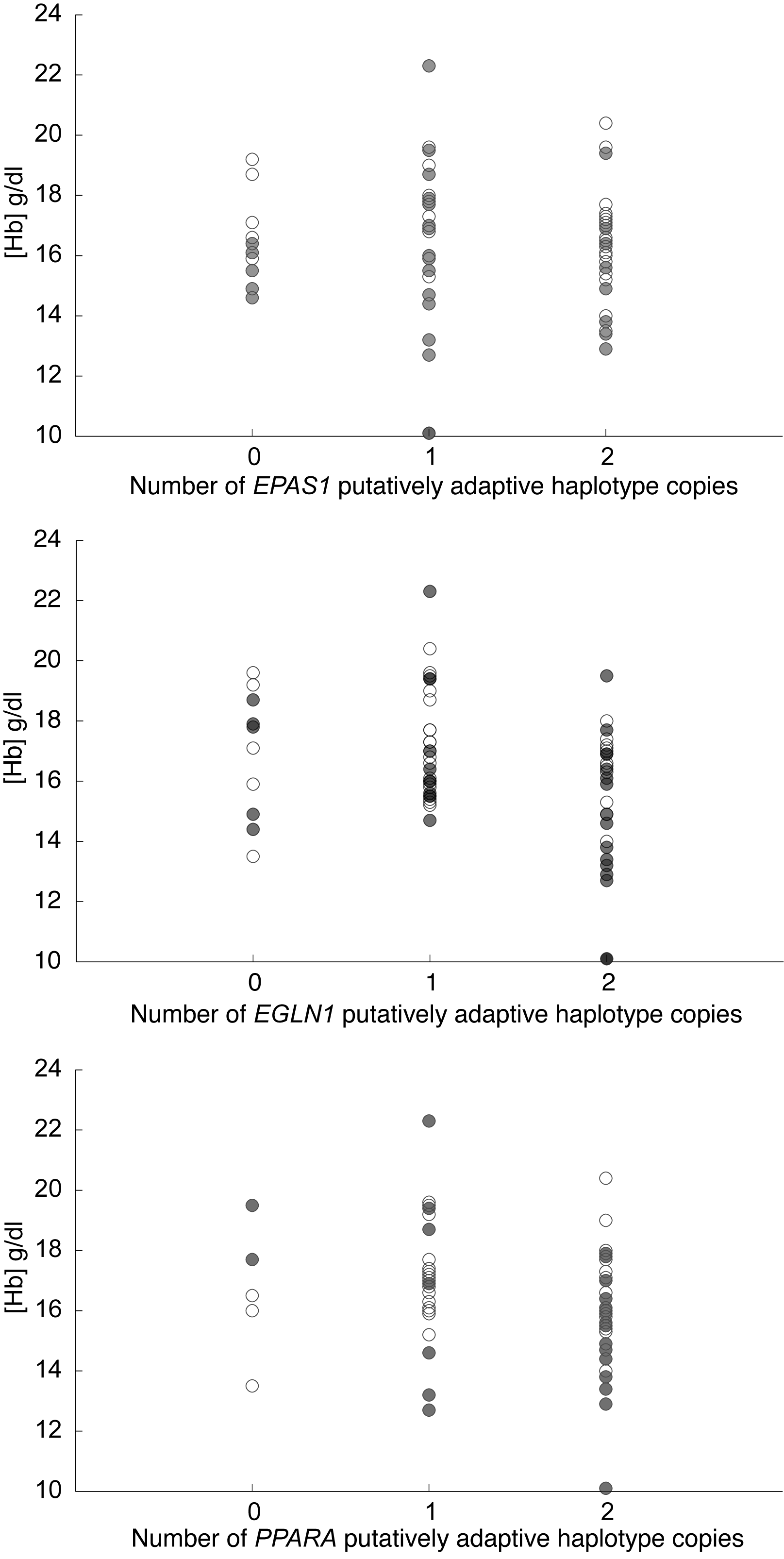

Supplement: Figure S1 — The relationships between EPAS1 , EGLN1 , and PPARA haplotypes and [Hb] for Tuo Tuo River Tibetans (shown in open circles) and Maduo Tibetans (closed circles). The number of PPARA haplotype copies, previously associated with [Hb] in Maduo Tibetans (p<0.0005; Simonson et al. 2010), is associated with [Hb] when data from both populations are combined (p<0.02). (TIF) [file pone.0088252.s001.tif]

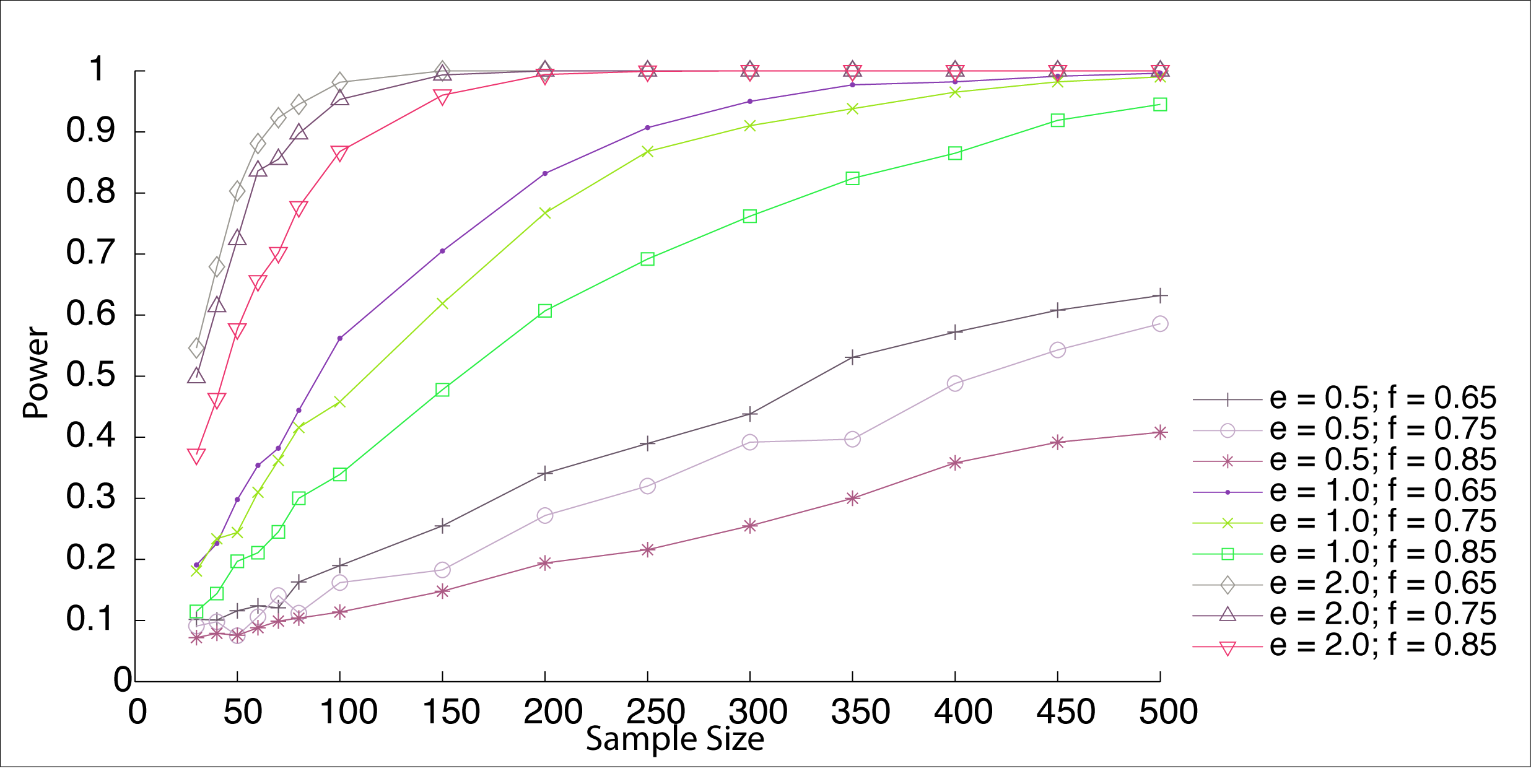

Supplement: Figure S2 — Statistical power to detect an association of [Hb] with a haplotype. Simulated data sets were constructed with varying sample size (n = 30–500), assuming that the putatively selected haplotype at one locus decreases [Hb] by e g/dl when present in two copies and e/2 if present in one copy (additive model, e = 0.5, 1.0, 2.0). [Hb] was simulated as a normally-distributed variable with mean 19.6 and standard deviation of 1.6 g/dl, as observed in the Tuo Tuo River sample, and the effect of adaptive haplotype copies was added to that variate for each individual. The frequency f of the adaptive haplotype was set at 0.65, 0.75 or 0.85, per the legend. To mirror the actual tests performed, haplotypes with no effect on [Hb] were simulated for two additional loci (haplotype frequency of 0.65 for both). Genotypes were assigned in Hardy-Weinberg equilibrium. Ages were assigned from a normal distribution, mean 37 years and standard deviation 11.5, then truncated to the range of 18–68, mirroring the observed distribution. Sex was assigned randomly with a 50/50 ratio. Multiple stepwise linear regression was performed using the five simulated predictors: age, sex and haplotype copies at three loci (as used in Table S3). Power to detect a significant association of [Hb] with the simulated adaptive haplotype was estimated as the fraction of 1000 iterations for each parameter set that yielded a significant result at the alpha = 0.5 level. Effect size e has the largest impact on statistical power. Haplotype frequency has a modest influence (Tuo Tuo River EGLN1, EPAS1, and PPARA frequencies = 0.68, 0.81, and 0.77, respectively). Considering our modest sample size, it will be necessary to collect more data from the Tuo Tuo River population to achieve greater power to detect genotype-phenotype associations. (TIF) [file pone.0088252.s002.tif]
