# Supplementary material for: Shared and Unique Signals of High-Altitude Adaptation in Geographically Distinct Tibetan Populations
Source: PLoS One. 2014 Mar 18;9(3):e88252. doi: 10.1371/journal.pone.0088252 (PMC3958363; doi:10.1371/journal.pone.0088252)
Supplement: Table S1 — FST for Tibetans and neighboring Asian populations examined. (DOCX) [file pone.0088252.s003.docx]

**Table S1.** F_ST_ for Tibetans and neighboring Asian populations examined

|  | Buryat Mongolian | HapMap Chinese (CHB) | HapMap Japanese (JPT) | Deedu Mongolian | TTR Tibetan |
| --- | --- | --- | --- | --- | --- |
| CHB | 0.016 |  |  |  |  |
| JPT | 0.017 | 0.007 |  |  |  |
| Deedu Mongolian | 0.008 | 0.013 | 0.016 |  |  |
| TTR Tibetan | 0.020 | 0.014 | 0.020 | 0.011 |  |
| MaduoTibetan | 0.017 | 0.012 | 0.018 | 0.013 | 0.004 |
