# Supplementary material for: Shared and Unique Signals of High-Altitude Adaptation in Geographically Distinct Tibetan Populations
Source: PLoS One. 2014 Mar 18;9(3):e88252. doi: 10.1371/journal.pone.0088252 (PMC3958363; doi:10.1371/journal.pone.0088252)
Supplement: Table S2 — Intersection of selection candidate regions and hypoxia-related miRNAs. (DOCX) [file pone.0088252.s004.docx]

**Table S2.** Intersection of selection candidate regions and hypoxia-related miRNAs

| Genes in region | Chromosome | 200kb bin | P value | miRNA | Population |
| --- | --- | --- | --- | --- | --- |
| *C21orf34* | Chr21 | 84 | 0.048 | mir125b2 and let7c | Maduo |
| *PPARA*,*C22orf26* | Chr22 | 224 | 0.009 | let7a3 | Maduo |
