# Supplementary material for: Shared and Unique Signals of High-Altitude Adaptation in Geographically Distinct Tibetan Populations
Source: PLoS One. 2014 Mar 18;9(3):e88252. doi: 10.1371/journal.pone.0088252 (PMC3958363; doi:10.1371/journal.pone.0088252)

**Table S3.** Multiple stepwise linear regression including age, sex, and three haplotypes previously identified as selection candidates in Tibetans (*EGLN1*, *PPARA*, and *EPAS1*) in Tuo Tuo River Tibetans. Gender was the only significant (p < 0.01) predictor in this analysis (F = 14.82, P < 0.0005).

| Predictor variable | P value | Effect size |
| --- | --- | --- |
| Age | 0.46 | -0.02 |
| Sex | 0.0005 | -1.79 |
| *EGLN1* | 0.80 | 0.10 |
| *PPARA* | 0.98 | 0.01 |
| *EPAS1* | 0.19 | -0.39 |

**Figure S1.** The relationships between *EPAS1*, *EGLN1*, and *PPARA* haplotypes and [Hb] for Tuo Tuo River Tibetans (shown in open circles) and Maduo Tibetans (closed circles). The number of *PPARA* haplotype copies, previously associated with [Hb] in Maduo Tibetans (p < 0.0005; Simonson et al. 2010), is associated with [Hb] when data from both populations are combined (p < 0.02).


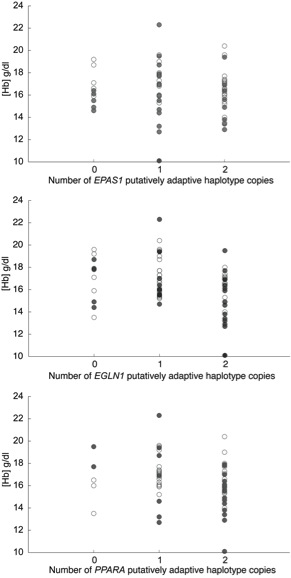


**Figure S2.** Statistical power to detect an association of [Hb] with a haplotype. Simulated data sets were constructed with varying sample size (*n* = 30-500), assuming that the putatively selected haplotype at one locus decreases [Hb] by *e* g/dl when present in two copies and *e*/2 if present in one copy (additive model, *e* = 0.5, 1.0, 2.0). [Hb] was simulated as a normally-distributed variable with mean 19.6 and standard deviation of 1.6 g/dl, as observed in the Tuo Tuo River sample, and the effect of adaptive haplotype copies was added to that variate for each individual. The frequency *f* of the adaptive haplotype was set at 0.65, 0.75 or 0.85, per the legend. To mirror the actual tests performed, haplotypes with no effect on [Hb] were simulated for two additional loci (haplotype frequency of 0.65 for both). Genotypes were assigned in Hardy-Weinberg equilibrium. Ages were assigned from a normal distribution, mean 37 years and standard deviation 11.5, then truncated to the range of 18-68, mirroring the observed distribution. Sex was assigned randomly with a 50/50 ratio. Multiple stepwise linear regression was performed using the five simulated predictors: age, sex and haplotype copies at three loci (as used in Supplementary Table 3). Power to detect a significant association of [Hb] with the simulated adaptive haplotype was estimated as the fraction of 1000 iterations for each parameter set that yielded a significant result at the alpha = 0.5 level. Effect size *e* has the largest impact on statistical power. Haplotype frequency has a modest influence (Tuo Tuo River *EGLN1*, *EPAS1*, and *PPARA* frequencies = 0.68, 0.81, and 0.77, respectively). Considering our modest sample size, it will be necessary to collect more data from the Tuo Tuo River population to achieve greater power to detect genotype-phenotype associations.


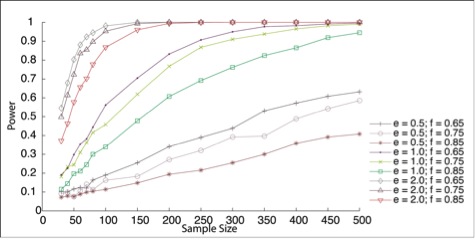

Supplement: Table S3 — Multiple stepwise linear regression including age, sex, and three haplotypes previously identified as selection candidates in Tibetans ( EGLN1 , PPARA , and EPAS1 ) in Tuo Tuo River Tibetans. Gender was the only significant (p<0.01) predictor in this analysis (F = 14.82, P<0.0005). (DOCX) [file pone.0088252.s005.docx]
